# Pregnancy Rates in Women With Polycystic Ovary Syndrome (PCOS) Using Letrozole Versus Clomiphene Citrate: A Retrospective Record Review

**DOI:** 10.7759/cureus.42257

**Published:** 2023-07-21

**Authors:** Yara O Bahawi, Ebtesam M Radwan, Maryam A Khouj, Rahaf K Alotaibi, Nada A Bajuwaiber, Lama F Baghlaf, Wala F AlFaraj, Ayman M Oraif

**Affiliations:** 1 Department of Medicine, King Abdulaziz University Faculty of Medicine, Jeddah, SAU; 2 Department of Obstetrics and Gynecology, King Abdulaziz University, Jeddah, SAU

**Keywords:** infertility drugs, clomiphene citrate, letrozole, pregnancy rates, polycystic ovary syndrome (pcos)

## Abstract

Background and objectives

Polycystic ovary syndrome (PCOS) is a prominent cause of anovulation. Thus, this study aimed to compare the pregnancy rates of women with PCOS treated with letrozole (LE) or clomiphene citrate (CC) at King Abdulaziz University Hospital.

Patients and methods

A retrospective record review was conducted from April 2021 to August 2022 to review 1370 records of women with PCOS from January 2015 to December 2021. Sixty-one patients were included in this analysis. Chi-square tests and independent sample t-tests were used to analyze various associations. Statistical significance was set at *P* < 0.05.

Results

Letrozole was associated with a higher pregnancy rate (41.7%) than CC (32.0%). However, this relationship was not statistically significant (*P* = .619). Furthermore, patients treated with letrozole required fewer cycles to achieve pregnancy (two cycles compared to three cycles). The different age groups and body mass indexes did not affect the pregnancy rate in either group.

Conclusion

No significant difference was found between CC and LE in ovulation induction and outcome among PCOS patients. Studies with larger sample sizes and multiple centers should be conducted in Saudi Arabia to obtain more conclusive results, which will eventually lead to changes in guidelines for anovulation treatment in women with PCOS.

## Introduction

Polycystic ovary syndrome (PCOS) is the most common endocrinopathy in women of childbearing age [1], with a prevalence of 6-26% worldwide [2-5]. It is a prominent cause of anovulation, a common problem in women, and is responsible for almost 40% of female infertility cases [6]. The Rotterdam criteria established in 2003 diagnose PCOS when at least two of the following three characteristics are present: menstrual irregularities or anovulation, clinical symptoms (acne, excess hair growth, or male pattern balding), or biochemical evidence of increased androgen levels (hyperandrogenism), and polycystic ovaries detected on pelvic ultrasonography, in addition to ruling out other conditions with similar symptoms [5,7,8].

Many ovulation induction plans have been proposed to increase ovulation rates in infertile women with anovulatory PCOS [9]. For example, clomiphene citrate (CC) is a selective estrogen receptor modulator commonly used as a first-line medication to induce ovulation in PCOS patients, with up to an 80% ovulation rate in previously anovulatory women [10-13]. However, CC has multiple disadvantages.

The anti-estrogenic effect of CC causes an extended depletion of estrogen receptors, which may cause peri-menopausal symptoms, including hot flashes and changes in cervical mucus features [14,15]. Moreover, 20-25% of PCOS patients are resistant to CC and thus cannot be treated with it [16,17]. Furthermore, although ovulation rates are high after treatment with CC, pregnancy rates following the treatment are relatively low at 30-40%, and this, paired with side effects such as multi-follicular formation and cyst development, are areas of concern regarding the use of the drug [6,18]. Miscarriage rates following pregnancy after treatment with CC are also higher than those in the general population [19,20]. Generally, although ovulation rates increase noticeably with the use of CC, the outcome of a successful pregnancy remains uncertain [17].

In CC-resistant women, letrozole (LE) is used as a second-line treatment option [16,21]. LE is a third-generation aromatase inhibitor widely used to treat breast cancer [22]. It increases the secretion of follicle-stimulating hormone (FSH) and luteinizing hormone (LH) from the anterior pituitary gland by inhibiting the enzyme responsible for the androgen-to-estrogen conversion, which would otherwise inhibit the hypothalamic-pituitary axis. This increase in FSH and LH levels leads to the growth of ovarian follicles [23-25], theoretically, increases pregnancy rates [26]. As LE does not directly influence estrogenic receptors like CC, it is devoid of anti-estrogenic adverse effects targeting the endometrium or cervical mucus quantity and quality [16,27]. Moreover, accumulated androgens in the ovaries increase the sensitivity of the follicles to FSH [24]. LE is now gaining popularity because of its advantages over CC and its multiple clinical uses [28,29].

LE is more sensitive to ovulation induction than CC, particularly in CC-resistant women [30]. It has a relatively shorter half-life (48 h) than CC and is therefore cleared more readily from the body [31,32], lowering the possibility of periconceptional exposure [26]. Furthermore, it is associated with fewer chances of multiple (twin) pregnancies through mono-follicular recruitment [27,33,34]. LE can cause side effects that are similar to menopause, such as hot flashes, difficulty sleeping, fatigue, depression, and vaginal dryness. Typically these symptoms get better within the first few months. Other common side effects include hair loss, muscle pain, bone pain, fractures, sweating, nausea, diarrhea, and dizziness [35].

Multiple studies have demonstrated the superiority of LE over CC in ovulation, pregnancy, and live birth rates [36-38]. However, the use of LE as first-line therapy for anovulation in PCOS continues to be debated [16,21]. In 2020, Sakar and Oglak conducted a retrospective study in Turkey to compare the clinical outcomes of ovulation induction by timed intercourse between LE and CC [39]. The results showed that patients in the LE group had higher pregnancy rates (52%) than those in the CC group (41.2%). A double-blind, multicenter randomized trial conducted in the USA showed that the live birth rate ratio of LE to CC was 1.44 (27.5%:19.1%). It was statistically proven that ovulation and pregnancy rates were higher in the LE group, with p-values of < 0.01 and 0.03, respectively. Both studies recommended the use of LE over CC in women with PCOS.

Although LE has shown higher pregnancy rates, as shown in the studies mentioned above, its use as a first-line treatment over CC remains controversial. This field of science has not been deeply researched in Saudi Arabia, and a few retrospective studies have addressed this valuable topic globally. This study aimed to determine whether LE or CC is best for increasing pregnancy rates in women with PCOS at King Abdulaziz University Hospital (KAUH), Jeddah, Saudi Arabia. The secondary objective was to demonstrate the effect of age and body mass index (BMI) on pregnancy rates with the use of both drugs from April 2021 to August 2022. The results of this study will significantly help infertility consultants manage anovulatory women with PCOS.

## Materials and methods

Study design, setting, and research ethics

A retrospective record review was conducted from April 2021 to August 2022 for all women diagnosed with PCOS (ICD-10 code: E28.2) and treated at KAUH, Jeddah, Saudi Arabia, with either LE or CC to achieve pregnancy. This study was approved by the Institutional Review Board of KAUH (Ref. 274-21) to determine the efficacy of LE compared to CC in achieving pregnancy in anovulatory women diagnosed with PCOS.

Study participants

Patients aged 18-45 years with a healthy uterine cavity and at least one patent fallopian tube were included in this study. However, patients with a BMI greater than 40 kg/m^2^, untreated thyroid diseases, untreated hyperprolactinemia, or patients treated for breast or ovarian cancer using LE were excluded.

Data collection and sample size

Using the hospital’s inpatient and outpatient pharmacy archives, we obtained the medical records of all patients who used LE and CC from January 2015 to December 2021. A total of 1370 medical records were filtered, removing patients treated with these agents for other medical indications and PCOS patients who did not meet the study’s inclusion criteria. Therefore, we obtained a convenience sample of 61 patients.

Data collected from the medical records included the following variables: a diagnosis of PCOS, age, BMI, thyroid-stimulating hormone (TSH) level, the medication used for the treatment of any thyroid dysfunction (if present), prolactin level, the medication used for the treatment of hyperprolactinemia (if present), the medication used to achieve pregnancy (LE or CC), and beta-human chorionic gonadotropin level or the presence of pregnancy follow-ups after the treatment. In addition, if pregnancy was achieved, the number of treatment cycles required to achieve pregnancy and the presence of single or multiple pregnancies were also obtained. In this study, normal TSH level was defined as 0.27-4.2 mIU/L. Normal prolactin levels were defined as less than 25 ng/mL for non-pregnant women and 80-400 ng/mL for pregnant women [39]. All the above data were collected based on the time of drug administration. Patients’ contact numbers were also recorded and used to contact them in case of missing data in the hospital records or if there was a loss of follow-up. The missing data varied depending on what was missing from the patient record. This included treatment cycles, pregnancy outcomes (whether or not the pregnancy was achieved), and whether the pregnancy was single or multiple. This is due to the continuity of perinatal care of the patients in other hospitals.

Data entry and statistical analysis

Microsoft® Excel data collection sheet version 2022 (Microsoft® Corp., Redmond, WA, USA) was used to enter the data from the medical records. The diagnosis of PCOS was based on ICD-10 codes, medical progress notes written by the patients’ gynecologists, and radiological imaging reports confirmed by the patients’ physicians.

Statistical Package for Social Sciences (SPSS) version 26 (IBM Corp., Armonk, NY, USA) was used to analyze the data. To describe the research, the central tendency and standard deviation were calculated for continuous variables. For categorical variables, frequencies were used to illustrate the numbers and percentages. All values in this study were written in the form of mean [standard deviation]. Independent samples t-tests and Chi-square tests were used to evaluate bivariate relationships. Statistical significance was set at p < 0.05.

## Results

Demographics and sample distribution

A total of 1370 records were reviewed, of which 61 patients who fulfilled the study’s inclusion criteria were included in the analysis. We divided our patients based on their prescribed medication: group 1 included 36 patients who used LE, and group 2 included the remaining 25 patients who used CC. The two groups were not matched for age, BMI, or medical background. The comparison of pregnancy rates between the two groups was based on the use of the two drugs. The average age of our sample was 31.2 [4.9] years, with a BMI of 26.6 [5.0] kg/m^2^.

Pregnancy rate

LE users achieved pregnancy in 41.7% (n = 15) of the cases, with 1.8 [0.8] cycles of treatment. On the other hand, 32.0% (n = 8) of the women treated with CC achieved pregnancy, requiring 2.6 [1.2] treatment cycles. Although LE had a higher pregnancy rate, the relationship between the type of drug used and the pregnancy rate was insignificant (P = .619).

**Figure 1:**
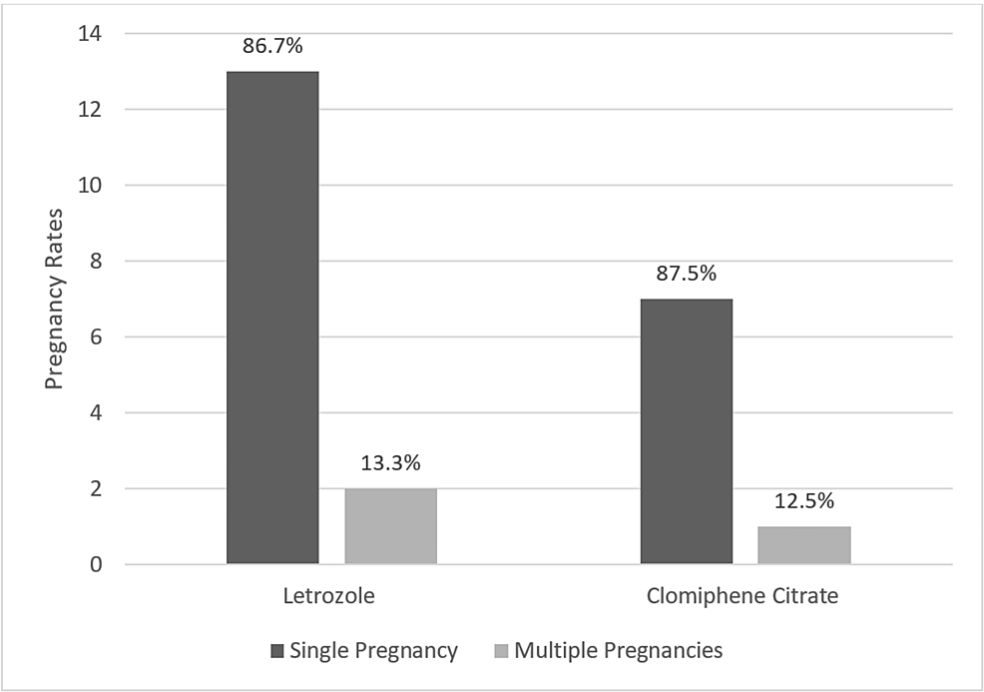
Effect of letrozole and clomiphene citrate on the pregnancy rates and outcomes of women with polycystic ovary syndrome

Regarding whether the women achieved single or multiple pregnancies, 13.3% (n = 2) of the women treated with LE had multiple pregnancies. In comparison, those treated with CC had multiple pregnancies in 12.5% (n = 1) of cases. Furthermore, the Chi-square test used to compare the effect of both drugs on the number of pregnancies revealed no statistically significant difference between the two drugs (P = .731), as demonstrated in Figure 1.

Age and BMI

To demonstrate the effects of age and BMI on the response to medications, we used an independent sample t-test to determine the impact of age and BMI on the pregnancy rate. Patient age and BMI did not affect the pregnancy rate in either the LE or CC group, as shown in Table 1.

**Table 1:** Effect of age and body mass index on achieving pregnancy with letrozole or clomiphene citrate. *Mean [standard deviation]. P: Pregnancy was achieved on the medication. NP: Pregnancy was not achieved on the medication. Total N = 61. No missing data.

Variables	Letrozole (N = 36)			Clomiphene citrate (N = 25)		
	Group	Value*	P-value	Group	Value*	P-value
Age (years)	P (n = 15)	30.0 [5.4]	0.260	P (n = 8)	28.9 [4.7]	0.093
	NP (n = 21)	31.9 [4.5]		NP (n = 17)	32.4 [4.8]	
Body mass index (kg/m^2^)	P (n = 15)	26.7 [4.4]	0.629	P (n = 8)	26.1 [6.4]	0.501
	NP (n = 21)	25.9 [5.0]		NP (n = 17)	27.8 [4.8]	

## Discussion

In our study, LE had a significantly higher pregnancy rate than CC. Similar results were obtained in an Indian clinical trial by Bansal et al. [40], in which the pregnancy rate in the LE group was almost double that of the CC group, and in a Pakistani study that also reported a 10% higher occurrence of pregnancy in LE than in CC [39]. Both previous studies recommended that LE be used as a first-line treatment for anovulation in PCOS patients due to the higher pregnancy and live birth rates it was associated with, similar to a third study by Amer et al. reporting elevated pregnancy and live birth rates in patients who were treated with LE compared to those treated with CC [39-41].

A Chinese study by Liu et al., on the other hand, concluded that LE did not improve overall pregnancy rates and pregnancy outcomes in PCOS patients, despite achieving higher ovulation rates, and recommended that CC be continued as a first-line agent in treating infertility due to anovulation in PCOS patients [42]. Another piece of literature published in Saudi Arabia also concluded that the effect of LE on ovulation and pregnancy is comparable to that of CC but not necessarily better, as the pregnancy rate is only slightly higher among women treated with LE versus those treated with CC [43]. Similarly, an Iranian study detected an increase in the number of pregnancies with LE compared to CC but found no statistically significant difference between the two and concluded that both drugs were effective, but neither was superior [44]. The pregnancy rates differed significantly from one study to the other, which may be attributed to the different characteristics of participants in each piece of literature, including different ethnicities, BMIs, age groups, and definitions of pregnancy (biochemical or clinical).

As for the number of treatment cycles our participants underwent before getting pregnant, it took almost twice the number of cycles to achieve pregnancy with CC than with LE. Similar deductions were reported by Legro et al., in which 750 women were divided in a 1:1 ratio to receive LE or CC for up to five treatment cycles. Pregnancy per treatment cycle was higher among women treated with LE than those treated with CC [34]. Amer et al. showed comparable results, as the median number of treatment cycles until pregnancy was much smaller with LE than with CC [41]. This may be explained by the research showing higher endometrial receptivity and endometrial thickness under the effect of LE, which may lead to earlier pregnancy [45].

In our study, 13.3% and 12.5% of women who achieved multiple pregnancies on medication were on LE and CC, respectively. This is contrary to the studies by Bansal et al. and Legro et al., in which CC was associated with multiple pregnancies [34,40]. The small sample size in our study compared to the other two studies, and thus the limited generalizability of our results, may have been attributed to the different statistics here.

Age and BMI were not significantly associated with pregnancy rates in this study between the groups (p > 0.05). Comparable results were detected in an Egyptian study that reported no significant difference between LE responders and non-responders regarding age, period of infertility, or BMI [46]. Another Egyptian study from 2019 found no significant association between the occurrence of pregnancy with either medication and the participants’ ages or BMIs [47]. A retrospective study conducted in Oman investigated the effect of LE combined with FSH on obese and non-obese women in achieving pregnancy and did not find a significant comparison [48]. Similar to our findings, a study conducted at Al-Azhar University Hospital in Egypt detected higher pregnancy rates with LE versus CC, regardless of age. Contrary to our findings, however, statistically significant associations were found with BMIs < 30 kg/m^2^, with pregnancy rates significantly better in the LE group than in the CC group, while no statistically significant difference in pregnancy rate was detected in women with BMIs > 30 kg/m^2^ [49].

Strengths and limitations

The limitations of this study include its retrospective design and the small sample size. A prospective study would enable researchers to monitor participants’ treatment journeys more effectively. Furthermore, tracking FSH levels, ovulation, and endometrial thickness would yield more precise information regarding the effects of both drugs.

Our study included 61 patients, which is more than the sample sizes in previous studies conducted in our region. Moreover, contacting the patients via their mobile numbers to complete the missing information in their medical records led to the absence of any missing data due to loss of follow-up or inadequacy of medical records, which is a limitation of many retrospective studies.

## Conclusions

Our study showed a higher pregnancy rate and a quicker time to pregnancy with LE as an ovulation inducer in PCOS patients than CC. More research comparing both treatment options is needed to properly study the effect of both drugs on ovulation induction and pregnancy in PCOS patients throughout the country.
